# Mesophyll conductance and cell wall composition: insights from a meta‐analysis across species

**DOI:** 10.1111/nph.70024

**Published:** 2025-02-27

**Authors:** Coralie E. Salesse‐Smith, Yi Xiao

**Affiliations:** ^1^ Carl R. Woese Institute for Genomic Biology University of Illinois at Urbana‐Champaign Urbana IL 61801 USA; ^2^ Pacific Northwest National Laboratory Richland WA 99354 USA

**Keywords:** carbon dioxide, cell wall composition, mesophyll conductance, meta‐analysis, pectin

## Abstract

This article is a Commentary on Roig‐Oliver *et al*. (2025), **246**: 2384–2391.

Mesophyll conductance (*g*_m_) plays a critical role in plant photosynthesis by regulating the diffusion of CO_2_ from substomatal cavities to the site of carbon fixation within the chloroplasts. Despite its importance, our understanding of the factors influencing *g*_m_, particularly the role of cell wall properties, remains incomplete. In this issue of *New Phytologist*, Roig‐Oliver *et al*. (2025; pp. 2384–2391) address this knowledge gap through a comprehensive meta‐analysis across multiple species from the major clades of land plants. By analyzing trends in *g*_m_ and two other key physiological parameters – the ratio of pectin to cellulose and hemicellulose in the cell wall by weight (P/(C + H)), and cell wall thickness (*T*_cw_) – their findings reveal strong correlations of *g*_*m*_ with the combination of P/(C + H) and *T*_cw_, where *g*_m_ is directly proportional to P/(C + H) and inversely proportional to *T*_cw_. This work not only underscores the influence of cell wall composition on CO_2_ diffusion but also provides a valuable framework for further experimental and modeling studies. By shedding light on the dynamic properties of cell walls, shaped by both composition and environmental regulation, this study opens new frontiers for understanding and improving photosynthesis.

*This highlights the potential of cell wall composition as a measurable proxy for effective porosity, advancing our understanding of the variability of* g_*m*_ *across species.*

Mesophyll conductance can be dissected into several components, representing diffusion barriers in the intercellular airspace, the cell wall, the plasma membrane, the cytosol, the chloroplast envelope and the chloroplast stroma. Among these, the cell wall has been generally considered the largest barrier to CO_2_ diffusion, in line with the dominant role of cell wall thickness (*T*_cw_) in limiting *g*_m_, as demonstrated by the anatomical limitation analysis introduced by Tomás *et al*. (2013). Evans *et al*. (2009) proposed a conceptual model for cell wall conductance (*g*_cw_) based on CO_2_ diffusion following Fick's first law. In this model, cellulose and hemicellulose form a fibrous network that influences the porosity and tortuosity of the cell wall. The model defines *g*_cw_ as

(Eqn 1) $$g_{\mathrm{cw}}=\frac{D_{c}\times p}{T_{\mathrm{cw}}\times \tau }$$

where *D*_c_ is the diffusivity of CO_2_ in water, p is the cell wall porosity, and τ is the tortuosity of the CO_2_ diffusion path through the cell wall. Earlier studies, such as those by Tosens *et al*. (2012) and Tomás *et al*. (2013), demonstrated that accounting for *T*_cw_ improved predictions of *g*_m_ variation. However, these studies did not investigate the influence of p or τ on *g*_m_, due to difficulties in measuring and estimating these quantities. Roig‐Oliver *et al*. add a new layer of insight, showing that incorporating P/(C + H), which approximates the effective porosity (p/τ) factor in Eqn 1, further strengthens correlations between *g*_m_ and cell wall properties. This highlights the potential of cell wall composition as a measurable proxy for effective porosity, advancing our understanding of the variability of *g*_m_ across species.

Pectin influences cell wall conductance by modulating pore size and the hydrophilic nature of the cell wall matrix. Pectin's hydrocolloid properties enhance effective porosity (p/τ) to water and CO_2_. The degree of pectin methylesterification and subsequent de‐esterification play a critical role in altering cell wall stiffness and porosity. The effects of de‐esterification are determined by two mechanisms: blockwise and nonblockwise de‐esterification. Blockwise de‐esterification facilitates cross‐linking with Ca^2+^, leading to gelation and increased cell wall stiffness, while nonblockwise de‐esterification reduces cross‐linking, enhancing porosity and softness. This dynamic process is influenced by factors such as pH, cation concentration and the activity of pectin‐remodeling enzymes. Overall, pectin instigates a complex and responsive system within the cell wall, significantly impacting its mechanical and functional properties. For a more detailed and comprehensive review on this topic, readers are referred to Flexas *et al*. (2021).

Despite our current understanding of the complexity of pectin's influence on cell wall conductance, the relationship between cell wall composition and *g*_m_ still requires deeper exploration. Experimental measurements of these interactions, particularly *in vivo*, remain challenging. Computational modeling may offer a promising avenue for hypothesis generation and testing. For example, from the shift of an exponential decay between *g*_m_ and *T*_cw_ to a linear relationship between *g*_m_ and 1/*T*_cw_ × P/(C + H), effective porosity is one potential explanation. However, how other anatomical components, such as the plasma membrane and chloroplast stroma, influence *g*_m_ beyond the cell wall properties in Eqn 1, and whether these effects explain differences among plant groups, could become an immediate research question for modeling to explore. For example, fig. 2 from Roig‐Oliver *et al*. reports that mosses exhibit wide variation in both *T*_cw_ and P/(C + H), yet their 1/*T*_cw_ × P/(C + H) and *g*_m_ show relatively small variation, raising questions about the underlying mechanisms. Additionally, crops deviate from these regressions, suggesting that they may be governed by different mechanisms. Developing models that account for these dynamics could provide critical insights into the mechanistic basis of *g*_m_ sensitivity and help bridge the gap between cell wall properties and photosynthetic efficiency. Furthermore, such models could also provide insights into how cell wall conductance may respond to environmental conditions such as light, CO_2_, temperature and drought.

Another complication to understanding the relationship between cell wall composition and conductance to CO_2_ lies in the distinction between type I and type II primary cell walls, which differ in their composition. Type I cell walls are found in eudicot angiosperms, noncommelinoid monocot angiosperms and all gymnosperms, whereas type II cell walls are only found in the commelinoid monocot division of angiosperms, which includes key crops such as wheat and rice. Type I cell walls generally contain less pectin (10% vs 35%) and more aromatic molecules compared to type II walls. In addition, proteins comprise only 1% of mass in type II cell walls, in contrast to 10% in type I walls. Differences in hemicelluloses further distinguish the two types, with type I walls containing more xyloglucans, which play a key role in tightening and loosening the cell wall. These compositional differences underscore the importance of considering the type of cell wall (I vs II) when targeting the cell wall to improve CO_2_ diffusion and mesophyll conductance. This is particularly relevant because some of our most important crops are commelinoid monocots. A deeper understanding of the structural and functional differences between primary cell wall types is pivotal for advancing efforts to improve crop yield.

Roig‐Oliver *et al*. suggest that crops may be outliers in how cell wall composition affects photosynthetic traits, potentially due to indirect selection for higher *g*_m_ during breeding for improved performance. For instance, a comparative study between an elite soybean cultivar LD11 and four ancestral cultivars found that *g*_m_ in the elite cultivar was double that of its ancestral cultivars (Pelech *et al*., 2025). The study also revealed that improvements in *g*_m_ were smaller than improvements in photosynthetic biochemistry, suggesting the potential for further *g*_m_ improvements to benefit crop productivity. Together, these findings highlight the importance of studying both crop and noncrop species to gain a more comprehensive understanding of how cell wall composition affects *g*_m_ and photosynthetic performance in order to identify traits that could be exploited for crop improvement.

Looking ahead, expanding research across the phylogeny of land plants will be critical for bridging the gap between the mechanistic understanding of mesophyll conductance and photosynthetic efficiency. Exploring natural variation in cell wall composition, as well as leveraging gene editing and transgenic approaches, offers promising strategies to enhance photosynthesis and yield. Recent successes, such as manipulating cell wall thickness and effective porosity (Pathare *et al*., 2024; Salesse‐Smith *et al*., 2024), demonstrate the potential of these approaches. Moving forward, a major challenge will be to understand how cell wall composition interacts across plant tissues, developmental stages and in response to different environmental conditions. For example, recent work by Sun *et al*. (2025) examined how drought conditions influence cell wall properties and mesophyll conductance in cotton. Techniques like glycome profiling, which provides detailed information about cell wall structure and carbohydrate components beyond P/(C + H), will play a key role in addressing these challenges. Future developments in this field will require interdisciplinary research that integrates plant physiology, biophysics, computational modeling and advanced analytical techniques, all of which are essential for advancing our ability to optimize cell wall properties for improved photosynthesis and plant productivity under diverse environmental conditions.

## Disclaimer

The New Phytologist Foundation remains neutral with regard to jurisdictional claims in maps and in any institutional affiliations.
